# Prognostic Role of Ventricular Ectopic Beats in Systemic Sclerosis: A Prospective Cohort Study Shows ECG Indexes Predicting the Worse Outcome

**DOI:** 10.1371/journal.pone.0153012

**Published:** 2016-04-21

**Authors:** Giacomo De Luca, Silvia Laura Bosello, Francesca Augusta Gabrielli, Giorgia Berardi, Federico Parisi, Manuela Rucco, Giovanni Canestrari, Francesco Loperfido, Leonarda Galiuto, Filippo Crea, Gianfranco Ferraccioli

**Affiliations:** 1 Institute of Rheumatology and Affine Sciences - Department of Rheumatology, Catholic University of the Sacred Heart, Rome, Italy; 2 Division of Heart Failure and Cardiac Rehabilitation, Complesso Integrato Columbus, Rome, Italy; 3 Institute of Cardiology, Catholic University of the Sacred Heart, Rome, Italy; Nippon Medical School Graduate School of Medicine, JAPAN

## Abstract

**Background:**

Arrhythmias are frequent in Systemic Sclerosis (SSc) and portend a bad prognosis, accounting alone for 6% of total deaths. Many of these patients die suddenly, thus prevention and intensified risk-stratification represent unmet medical needs. The major goal of this study was the definition of ECG indexes of poor prognosis.

**Methods:**

We performed a prospective cohort study to define the role of 24h-ECG-Holter as an additional risk-stratification technique in the identification of SSc-patients at high risk of life-threatening arrhythmias and sudden cardiac death (SCD). One-hundred SSc-patients with symptoms and/or signs suggestive of cardiac involvement underwent 24h-ECG-Holter. The primary end-point was a composite of SCD or need for implantable cardioverter defibrillator (ICD).

**Results:**

Fifty-six patients (56%) had 24h-ECG-Holter abnormalities and 24(24%) presented frequent ventricular ectopic beats (VEBs). The number of VEBs correlated with high-sensitive cardiac troponin T (hs-cTnT) levels and inversely correlated with left-ventricular ejection fraction (LV-EF) on echocardiography. During a mean follow-up of 23.1±16.0 months, 5 patients died suddenly and two required ICD-implantation. The 7 patients who met the composite end-point had a higher number of VEBs, higher levels of hs-cTnT and NT-proBNP and lower LV-EF (p = 0.001 for all correlations). All these 7 patients had frequent VEBs, while LV-EF was not reduced in all and its range was wide. At ROC curve, VEBs>1190/24h showed 100% of sensitivity and 83% of specificity to predict the primary end-point (AUROC = 0.92,p<0.0001). Patients with VEBS>1190/24h had lower LV-EF and higher hs-cTnT levels and, at multivariate analysis, the presence of increased hs-cTnT and of right bundle branch block on ECG emerged as independent predictors of VEBs>1190/24h. None of demographic or disease-related characteristics emerged as predictors of poor outcome.

**Conclusions:**

VEBS>1190/24h identify patients at high risk of life-threatening arrhythmic complications. Thus, 24h-ECG-Holter should be considered a useful additional risk-stratification test to select SSc-patients at high-risk of SCD, in whom an ICD-implantation could represent a potential life-saving intervention.

## Introduction

Systemic Sclerosis (SSc) is a rare and life-threatening connective tissue disease characterized by diffuse vascular damage, aberrant activation of the immune system and fibrosis of skin and internal organs, associated with a high mortality risk [1]. Heart involvement is common during SSc and represents the leading cause of death in about one third of patients [1,2]. Cardiac involvement can be direct or indirect, i.e. related to pulmonary and renal involvement, and all cardiac structures may be involved, resulting in pericardial effusion, ventricular arrhythmias, conduction system defects, valve disease, myocardial ischaemia, myocarditis and heart failure [2]. Clinical presentation comprehends dyspnea, chest pain, palpitations and heart failure, although most patients are asymptomatic at early stages and the diagnosis is often delayed due to the lack of a specific diagnostic algorithm. Arrhythmias, in particular, are a frequent event and portend a bad prognosis. This latest notion dates back more than 30 years ago and was recently highlighted by data from Genetics Versus Environment In Scleroderma Outcome Study (GENISOS) cohort [3], reporting the poor prognostic meaning of clinically significant arrhythmias on electrocardiography (ECG); the dismal prognosis of Scleroderma heart disease and of its arrhythmic manifestations in particular, was further emphasized by a wide analysis of causes and risk factors for death in SSc from the EULAR Scleroderma Trials and Research (EUSTAR) database: myocardial involvement, indeed, accounted for 14% of SSc-related deaths, which were to a large part attributed to arrhythmia (6% of total deaths) [1].

Notably, previous studies reported that sudden cardiac death (SCD) accounts alone for about 5% of total deaths: in two large post-mortem analysis, SCD was the final event in 5% of SSc patients and was associated with ventricular arrhythmias and skeletal myositis [4,5]. Thus, its prevention is a major goal in the management of these patients. It is noteworthy that abnormal standard 12-lead ECG is present in 25–75% of SSc patients and is suggested as an independent predictor of mortality [6–8]. Furthermore, on 24h ECG-Holter, ventricular ectopy overall was common, occurring in 67% of SSc patients and was strongly correlated with both total mortality and sudden cardiac death in a prospective multicentre study dating back almost 30 years ago [6]. Notably, in this pioneering study both ventricular ectopic beats (VEBs) and SCD more likely occurred in patients with evidence of severe pulmonary involvement and pulmonary arterial hypertension (PAH); this is in line with the acquired knowledge that cardiac arrhythmias are important contributors to morbidity and mortality in patients with PAH and that SCD accounts alone for 28% deaths in these patients [9]. Conversely, the prevalence and the prognostic significance of ventricular arrhythmias in SSc patients with direct cardiac involvement, (i.e. unrelated to pulmonary disease), is not so well characterized and, in this clinical scenario, defined predictors of cardiac involvement and outcome are not available yet. Thus, intensified risk stratification is desperately needed to improve outcomes of SSc patients with heart involvement and to prevent SCD.

Taking into account the data of the old prospective study, never addressed and confirmed afterwards, the aim of our study was to clarify and define the role of 24h ECG-Holter in selected SSc-patients with signs and/or symptoms suggestive of direct cardiac involvement and its ability in identifying those at high risk of SCD or major arrhythmic complications. Importantly, our aim was achieved by the identification of a cut-off value of VEBs, emerged as prognostic relevant on ROC curve, and that could be considered the “warning biomarker” of major arrhythmic risk in selected SSc patients.

## Patients and Methods

One hundred selected SSc-patients with new onset of symptoms suggestive of cardiac involvement, such as dyspnea, palpitations, chest pain, and/or signs of heart failure (i.e., ankle edema, raised jugular pulse, pulmonary rales), unrelated to lung disease progression, and/or with an increase of cardiac enzymes, admitted from 2008 to 2013 to the outpatient clinic of the Rheumatology Division of our tertiary referral hospital, were enrolled in this prospective cohort study.

All patients fulfilled the criteria proposed by the American College of Rheumatology for SSc and were classified as having limited or diffuse disease according to LeRoy classification [10,11]. The extension of skin involvement was evaluated by the modified Rodnan skin score (mRSS; range from 0 to 51).

A comprehensive assessment of disease characteristics and organ involvement was performed and data on cardiovascular risk factors were available for all patient cohort; patients with history of coronary artery disease were excluded from the study (S1 Appendix)

All patients underwent standard 12-leads ECG and 24h-ECG-Holter monitoring by 12 channel digital recorders (Mortara Instrument Inc.^®^HScribe5^™^,Milwakee,USA). Rhythm alterations, presence and number of premature ventricular and supraventricular complexes (at least 100 per 24h), ventricular and supraventricular arrhythmias were considered ECG-Holter abnormalities [12]. In particular, frequent ventricular ectopic beats (VEBs) were defined as occurring ≥30/h [13], non-sustained ventricular tachycardia (NS-VT) was defined as ≥3 consecutive VEBs with a rate ≥100 beats/min terminating spontaneously in less than 30s, while sustained ventricular tachycardia (S-VT) was defined as any VT whose ventricular rate ranged from 160 beats/min to 210 beats/min lasting 30s and/or requiring termination due to hemodynamic compromise less than 30s [14]. Ventricular fibrillation (VF) was defined as a rapid, incessant, irregular ventricular rhythm >210 beats/min determining appropriate ICD shock [14].

All patients underwent two-dimensional (2D) echocardiographic Doppler examination. Left-ventricular ejection fraction (LV-EF) was measured on apical four- and two-chamber views using the Simpson method and pulmonary artery systolic pressure (PASP) was estimated using the tricuspid regurgitation velocity and the Bernoulli equation [15]. Twenty-three patients with signs and/or symptoms suggestive of PAH such as dyspnea, NT-proBNP raise and disproportionately reduced pulmonary diffusing capacity for carbon monoxide (DLCO) with FVC/DLCO ratio ≥ 1.82 [16], or with PASP >35 mmHg underwent right heart catheterization (RHC) to confirm PAH at study entry. A subsequent analysis based on the recently published DETECT prediction score, confirmed that none of the remaining 77 patients has to be referred to an RHC for confirmation of PAH [17].

Cardiac enzymes [high-sensitive cardiac troponin-T (hs-cTnT) and CK-MB], total creatine-phosphokinase (CPK) and NT-proBNP values were obtained in all patients within two weeks of 24h-ECG Holter monitoring. Dyspnea was categorized into four classes according to the New York Heart Association(NYHA) classification criteria [18].

Data obtained at time of 24h-ECG-Holter were used for the determination of length of follow-up and a mean follow-up of 23.1±16.0 months (range: 3–77 months) was reached. A composite outcome made of SCD or need for ICD-implantation was considered as the primary end-point. SCD was defined as “unexpected arrest of presumed cardiac origin within one hour after onset of any symptoms that could be interpreted as being cardiac in origin”, as previously reported [19]. Need for ICD implantation was based on current recommendations [14,20]. A subcutaneous ICD was used (SQ-RX^®^Pulse Generator, Model Number-1010^®^, Cameron Health Inc^®^, California,USA) and ICD-shocks were considered appropriate for S-VT or VF.

The presence of 24h ECG-Holter abnormalities was evaluated in a control group of 15 asymptomatic SSc patients without increase of cTnT and/or NT-proBNP and with normal echocardiographic findings (S1 Table).

The study is in agreement with the recommendations of Declaration of Helsinki and of the local Ethical Committee. The Catholic University Institutional Review Board approved the study and the written informed consent, which was obtained from each patient at time of study enrolment.

### Statistical analysis

Data were analyzed using SPSS15.0 (SPSS,Chicago,IL-USA). In the univariate analysis categorical variables were analyzed with chi-square test or Fisher’s exact test and continuous variables were assessed with the Mann-Whitney U test. Spearman’s rank correlation was used to correlate different disease parameters. Continuous variables are reported as mean±standard deviation (SD), while categorical as number and percentage.

To determine the best VEBs threshold capable of differentiating between patients who satisfied the primary end-point from those who did not, receiving operating characteristics (ROC) analysis was performed and the area under ROC curves (AUROC) was calculated; the optimal cut-point was chosen among the coordinates of the ROC curves at the point that gave a balanced weight between sensitivity and specificity [21].

A multivariate analysis was then performed to identify independent risk factors associated with SCD and VEBs>1190/24h. Variables were entered in a backward stepwise regression model, requiring an adjusted p value <0.1 to enter the next step of the analysis. For each variable in the final equation, odd ratio (OR), expressed as exp(B), where B is the coefficient of the variable in the logistic equation, 95% confidence interval (CI) and p value were reported. The Hosmer-Lemeshow test was used to assess the goodness of fit of the model. A p value <0.05 was considered statistically significant.

## Results

Demographic, clinical and immunological characteristics of 100 patients are summarized in Table 1. All enrolled patients presented symptoms and/or signs suggestive of cardiac involvement: fifty-eight (58%) experienced palpitations, while dyspnoea was present in 68 patients (68%). The majority of patients with dyspnea (82.3%) were in NYHA class II, while 12 (17.7%) were in NYHA class III-IV. Fourteen patients (14%) presented low-limbs edema, only 7 patients (7%) reported an history of sporadic and self-limited chest pain, while none had history of unexplained pre-syncope or syncope. Twenty-four (24%) had history of skeletal myositis, but only 3 had total CPK levels >190 UI/l in the two weeks before 24h-ECG Holter evaluation. Sixty-one patients (61%) had a modest but persistent increase of cardiac enzymes (hs-cTnT>0.014 ng/ml and/or CK-MB>4 ng/ml). Mean levels of hs-cTnT were 0.051±0.10 ng/ml and mean levels of CK-MB were 4.5±7.3 ng/ml. Fifteen patients (15%) had a diagnosis of PAH.

**Table 1:** Demographic, immunological and clinical characteristics of 100 selected SSc patients.

Characteristic	
Age, years (mean ± SD)	56.1 ± 15.2
Females, n(%)	85 (85)
Disease duration, years (mean ± SD)	10.2 ± 9.3
Diffuse disease, n(%)	55 (55)
Anti-Scl70 positivity, n(%)	46 (46)
Anti-Centromere positivity, n(%)	26 (26)
Interstitial lung involvement, n(%)	58 (58)
History of digital ulcers, n(%)	59 (59)
Patients with palpitations, n(%)	58 (58)
Patients with dyspnoea, n(%)	68 (68)
Dyspnoea NYHA II, n(%)	56 (82.3) *
Dyspnoea NYHA III-IV, n(%)	12 (17.7) *
Elevation of troponin T >0.014 ng/ml, n(%)	61 (61)
LV-EF (%),(mean ± SD)	59.9 ± 9.7
PASP, mmHg, (mean ± SD) **	33.2 ± 12.9
Pulmonary arterial hypertension on RHC	15 (15)
Total deaths†, n(%)	14 (14)
SCD, n(%)	5 (5)
Implantable Cardioverter Defibrillator requiring, n(%)	2 (2)
SCD/ICD, n(%)	7 (7)

SD: standard deviation; n: number; NYHA: New York Heart Association; LV-EF: left ventricular ejection fraction on echocardiography; PASP: pulmonary arterial systolic pressure; SCD: sudden cardiac death; ICD: Implantable cardioverter defibrillator; RHC: right heart catheterization. SCD/ICD-insertion means composite end-point consisting of sudden cardiac death or implantation of implantable cardioverter defibrillator.

*percentage was calculated on patients with dyspnea;

** PASP measured on echocardiography;

^†^13 out of 14 deaths observed during the four-year follow-up were directly related to SSc and ten were directly related to cardiac involvement (arrhythmias or heart failure due to PAH).

### ECG findings

Sixty-eight patients (68%) presented an alteration on standard ECG; the most common finding was the presence of ST-T non-specific changes, recorded in 34 patients (34%), followed by conduction defects. In particular a complete or incomplete right bundle branch block (RBBB) was present in 19 patients (19%). VEBs were detected in 10 patients (10%) and 12 patients (12%) had supraventricular ectopic beats (SVEBs).

### ECG-Holter findings

Among the 100 SSc-patients with suspected heart involvement, 24h ECG-Holter abnormalities were present in 56 (56%). The majority of patients (96%) was on sinus rhythm. The presence of VEBs was common (42%) and 24 patients (24%) presented frequent VEBs, as previously defined; considering that all the patients with frequent ventricular ectopy had a number of VEBs>1000/24h, we referred to this number [4]. The mean number of VEBs was strikingly high (2046.1±6027.8 during 24h), with a maximum of 33615/24h. Among patients with VEBs, these were classified as polymorphic in 11 (26.2%). SVEBs>1000/24h were also frequent (19%), with a mean number of 798.9±1835.6 per day; runs of SVEBs were recorded in seventeen patients (17%). Fourteen patients (14%) presented episodes of paroxysmal supraventricular tachycardia, with a maximum of 36 beats, while 11 patients (11%) exhibited runs of non-sustained VT (NS-VT), the longest of 34 beats. No patients presented S-VT. These data are summarized in Table 2.

**Table 2:** ECG-Holter findings in our cohort of 100 selected SSc patients.

ECG-Holter characteristics	
Pts with any alteration on 24h ECG-Holter, n (%)	56 (56)
Sinus rhythm, n (%)	96 (96)
Phases of idioventricular rhythm, n (%)	2 (2)
Phases of bigeminal rhythm, n (%)	9 (9)
Atrial fibrillation, n (%)	4 (4)
I° degree atrio-ventricular block, n (%)	4 (4)
Right bundle branch block, n (%)	19 (19)
Left bundle branch block, n (%)	4 (4)
Mean HR, bpm (mean ± SD)	79.4 ± 10.2
Maximum HR, bpm (mean ± SD)	131.7 ± 23.3
Minimum HR, bpm (mean ± SD)	54.8 ± 11.2
SVEBs*, n/24h (mean ± SD)	798.9 ± 1835.6
VEBs^†^, n/24h (mean ± SD)	2046.1 ± 6027.8
Pts with VEBs, n (%)^§^	42 (42)
Pts with SVEBs, n (%)^§^	49 (49)
Pts with VEB>1000/24h, n (%)	24 (24)
Pts with SVEB>1000/24h, n (%)	19 (19)
Pts with polymorphic VEB, n (%)	11 (26.2)**
Supraventricular tachycardia, n (%)	14 (14)
Non sustained ventricular tachycardia, n (%)	11 (11)
QTc, ms (mean ± SD)	411.1 ± 33.4
QTc >440 ms, n(%)	11 (11)

Pts: patients; n: number; SD: standard deviation. HR = heart rate; bpm = beats per minute;

*SVEBs: number of supraventricular ectopic beats during 24h evalutation;

^†^VEBs: number of ventricular ectopic beats during 24h evaluation;

^§^patients with at least 100 VEBs or SVEBs during 24h;

**this percentage was calculated on patients with VEBs; QTc = corrected QT interval.

The number of SVEBs and VEBs correlated with hs-cTnT levels (R = 0.3, p = 0.001 and R = 0.4,p<0.001,respectively), CK-MB levels (R = 0.2,p = 0.03 and R = 0.28,p = 0.003,respectively) and NT-proBNP levels (R = 0.5,p<0.001 and R = 0.2,p = 0.03,respectively). Furthermore, the number of VEBs and SVEBs inversely correlated with LV-EF on echocardiography (R = -0.4,p<0.001 and R = -0.3, p = 0.002,respectively) and directly correlated with disease severity index (R = 0.3,p = 0.03 and R = 0.3,p = 0.002,respectively); the number of VEBs, but not SVEBs, directly correlated with the extension of skin involvement evaluated by the mRSS (R = 0.2,p = 0.03) and with the corrected QT interval duration (R = 0.2, p = 0.04). The number of SVEBs, but not VEBs, directly correlated with the disease activity index (R = 0.2, p = 0.04).

No correlations were found between Holter abnormalities, VEBs in particular, and other demographic, clinical and immunological parameters usually collected in SSc patients, such as disease duration, disease cutaneous subset, history or digital ulcers or active/recurrent digital ulcers, PAH on RHC or history of active myositis (p = ns for all correlations). A slightly higher prevalence of Scl70 positivity was found among patients with VEBS >1000 per day compared to patients without frequent VEBs (62.5% vs 40.8%, p = 0.05).

The contribution of traditional cardiovascular risk factors such as arterial hypertension or diabetes mellitus, smoking history, body mass index (BMI) and serum levels of total cholesterol, high-density-lipoprotein (HDL), fasting glucose and triglycerides (available for all patient cohort) seems to be marginal to the development of the Scleroderma arrhythmic outburst, since no correlations were found with Holter abnormalities (data not shown).

Among the 15 asymptomatic SSc patients, arrhythmic events on 24h Holter monitoring, as previously defined, were recorded only in 4 cases (26.7%) (p = 0.032), and in all patients were short runs of paroxysmal supraventricular tachycardia.

Interestingly, none of the 15 asymptomatic SSc patients of the control group had a number of VEBs >1000 per day and their mean number was extremely low (63.9 ± 136.6 during 24h) (p<0.001) (S1 Table).

### Predictors of major arrhythmic complications

Considering the time from baseline 24h-ECG Holter, follow-up data were available for the entire study cohort. During the follow-up, 14 deaths occurred (14%), and most of them (92.8%) were related to SSc disease progression. In particular, 10 were directly related to cardiovascular involvement (arrhythmic complications or cardiac failure due to PAH). Five patients with PAH NYHA-IV died of congestive heart failure, and these 5 patients were excluded in the subsequent statistical analysis to focus on the primary cardiac involvement in SSc. Thus, five patients (5%), without PAH on RHC, died of SCD. All patients who suffered SCD presented with VEB>1000/24 at baseline 24h-Holter evaluation (p<0.001). Moreover, during the follow-up period, two young patients underwent ICD-implantation; in particular, one 43-year patient with VEBs>1000/24h at baseline presented a rest episode of S-VT that required electrical cardioversion and subsequent ICD-implantation; after 3 weeks, analysis of the device revealed several episodes of S-VT, which were promptly reverted by the appropriate electrical shock delivery. During the subsequent 3 months, moreover, further episodes of S-VT, resistant to anti-arrhythmic drugs and catheter ablation, were recorded. An endomyocardial biopsy was performed in this patient and revealed a borderline myocarditis with extensive fibrosis (data not shown). Another 32-year patient, with 19158 VEBs/24h at baseline, underwent an electrophysiological study with induction of an episode of VF requiring electrical cardioversion and subsequent ICD-implantation. Thus, 7 patients (the youngest was 32 years old, the oldest 77 years old) met the pre-specified primary composite end-point of SCD or ICD-implantation. The mean time from baseline ECG-Holter to death or ICD-implantation was 13.2 ± 11.1 months.

Patients who met the composite end-point had a mean disease duration of 9.7±7.3 years and presented higher number of VEBs and SVEBs, higher levels of hs-cTnT, CK-MB and NT-proBNP, and lower LV-EF on echocardiography with respect to patients who did not develop major arrhythmic cardiac events (Table 3), while no differences in the occurrence of the composite arrhythmic endpoint were observed according to the cardiovascular risk-factors profile (data not shown). Interestingly, despite the huge difference in LV function between the two groups (mean LV-EF of 39.4% among the patients who met the primary end-point compared to 61.6% in the other), the LV-EF range in patients who met the primary end-point was wide (23–60%) and one of these patients presented a preserved LV-EF.

**Table 3:** Clinical characteristics of patients who met the primary end-point (SCD+ICD) compared with patients without major arrhythmic events.

Characteristics	7 patients who met the primary end point	93 patients without arrhythmic events	P
**Age (years), mean±SD**	53.3 ± 12.9	56.3 ± 15.4	0.5
**Disease duration (years), mean±SD**	9.7 ± 7.3	10.3 ± 9.4	0.9
**Anti-Scl70 positivity,n(%)**	4 (57.1)	42 (45.1)	0.4
**Diffuse disease, n(%)**	6 (85.7)	49 (52.7)	0.07
**Skin score, mean±SD**	14.1 ± 12.3	9.2 ± 9.1	0.2
**Disease Activity Index, mean±SD**	5.8 ± 2.1	2.8 ± 1.9	0.003
**Disease Severity Index, mean±SD**	12.5 ± 4.1	6.7 ± 4.3	0.006
**History of digital ulcers, n(%)**	7 (100)	52 (55.9)	0.016
**NT-proBNP (pg/ml), mean±SD**	6562.3 ± 6367.6	843.6 ± 2023.4	0.001
**Troponin T values (ng/ml), mean±SD**	0.22 ± 0.26	0.04 ± 0.07	<0.0001
**Troponin T >0.014 ng/ml, n(%)**	7 (100)	54 (58.1)	0.01
**CK-MB values (ng/ml), mean±SD**	16.9 ± 21.6	3.7 ± 4.4	0.002
**CK-MB > 4 (ng/ml), n(%)**	5 (71.4)	20 (21.5)	0.03
LV-EF (%)*, mean±SD	39.4 ± 13.8	61.6 ± 7.2	<0.0001
LV-EF <55%*, n(%)	6 (85.7)	12 (12.9)	<0.0001
**VEBs (n/24h), mean±SD**	9679.1 ± 10780.5	1471.6 ± 5172.3	<0.0001
**SVEBs (n/24h), mean±SD**	3163.8 ± 3626.0	644.7 ± 1573.7	0.007
**VEBs>1000/24h, n(%)**	7 (100)	17 (18.3)	<0.0001
**SVEBs>1000/24h, n(%)**	4 (57.1)	15 (16.1)	0.01
**Right bundle branch block, n(%)**^†^	4 (57.1)	15 (16.1)	0.02
**Left bundle branch block, n(%)**	1 (14.2)	3 (3.2)	0.2
**NSVT, n(%)**	4 (57.1)	7 (4.3)	0.001
**QTc (ms), mean±SD**	431.6 ± 50.0	409.5 ± 31.6	0.2
**QTc >440 ms, n(%)**	2 (28.6)	9 (9.7)	0.2
**Palpitations, n(%)**	6 (85.7)	52 (55.9)	0.06
**Dyspnea, n(%)**	6 (85.7)	63 (67.7)	0.3

SCD = sudden cardiac death; ICD = implantable cardioverter defibrillator; SD = standard deviation; n = number; LV-EF: left ventricular ejection fraction; VEBs = ventricular ectopic beats; SVEBs = supraventricular ectopic beats; NSVT = non-sustained ventricular tachycardia;

*left ventricular ejection fraction on echocardiography;

^†^complete right bundle branch block on 24h ECG Holter.

**Fig 1:**
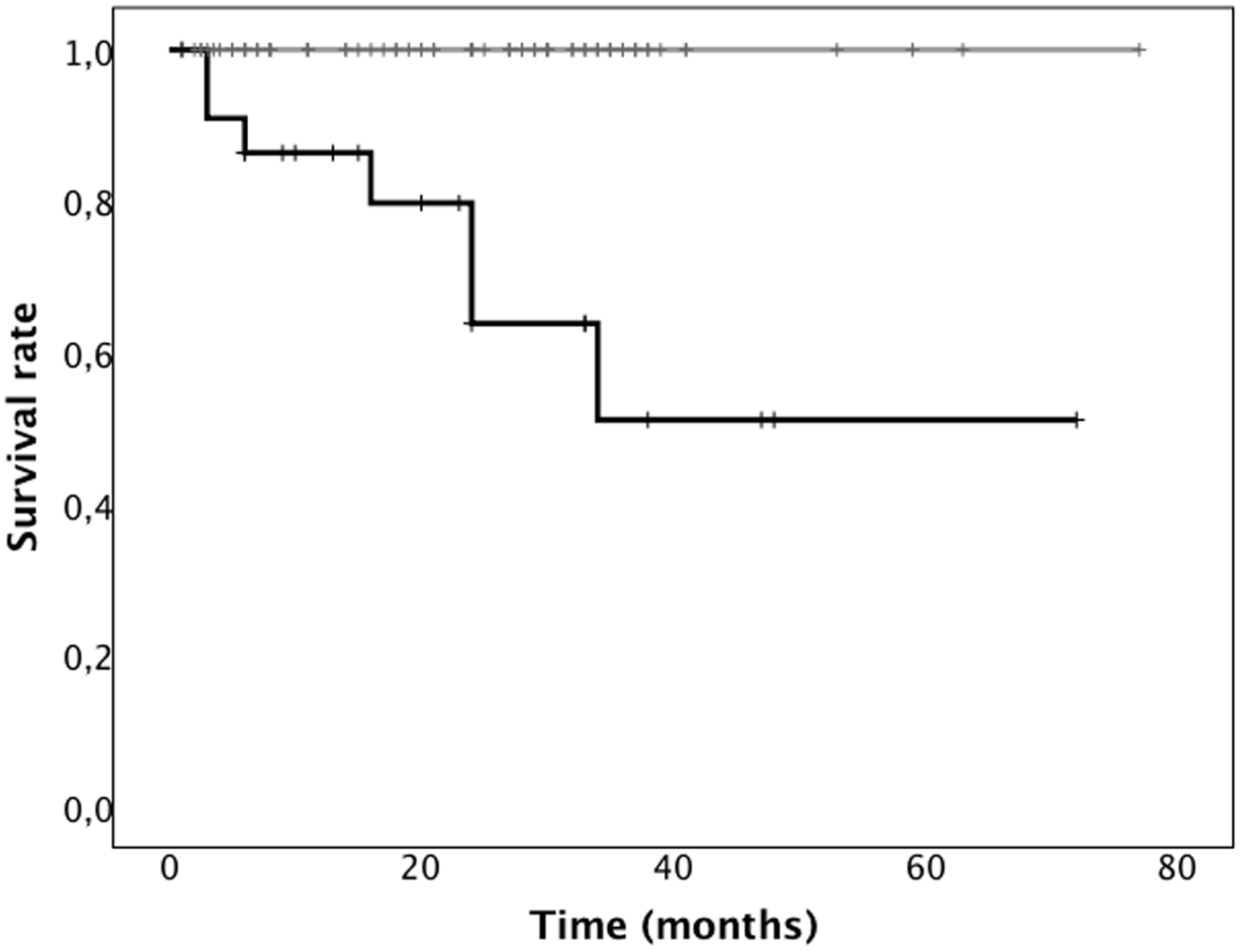
Survival curve in patients with and without VEBs>1000/24h. Kaplan-Meier estimates cumulative survival between patients with or without VEBs>1000/24h. Black line: survival in patients with VEBs>1000/24h. Grey line: survival in patients with VEBs<1000/24. Each tick mark corresponds to a time of patient censoring. None of the patients without VEBs <1000/24h died or required and ICD-implantation during follow-up while 7 patients with VEBs >1000/2h at baseline met the composite end-point SCD/ICD implantation.

All 7 patients who met the primary end-point had VEBs>1000 24/h at baseline 24h-ECG Holter, increased levels of hs-cTnT and NT-proBNP and history of digital ulcers. None of the patients without frequent VEBs died or presented major arrhythmic complications (Fig 1).

The composite end point (SCD+ICD) was associated with LV-EV <55% (RR:45.6,CI:4.9–424.4,p = 0.01) and presence of RBBB (RR:5.9,CI:1.2–29.9,p = 0.03), NSVT (RR:18.1,CI:2.6–127.2,p = 0.004) or SVEBs >1000/day on 24h-Holter monitoring (RR:7.1,CI:1.4–36.2,p = 0.02), after correction for demographical characteristics (age and sex).

None of the variables emerged as independent predictor of SCD on multivariate analysis, probably because of the limited sample size of our cohort and the small number of registered events.

### ROC analysis curve: VEBs are red flag of arrhythmic complications

**Fig 2:**
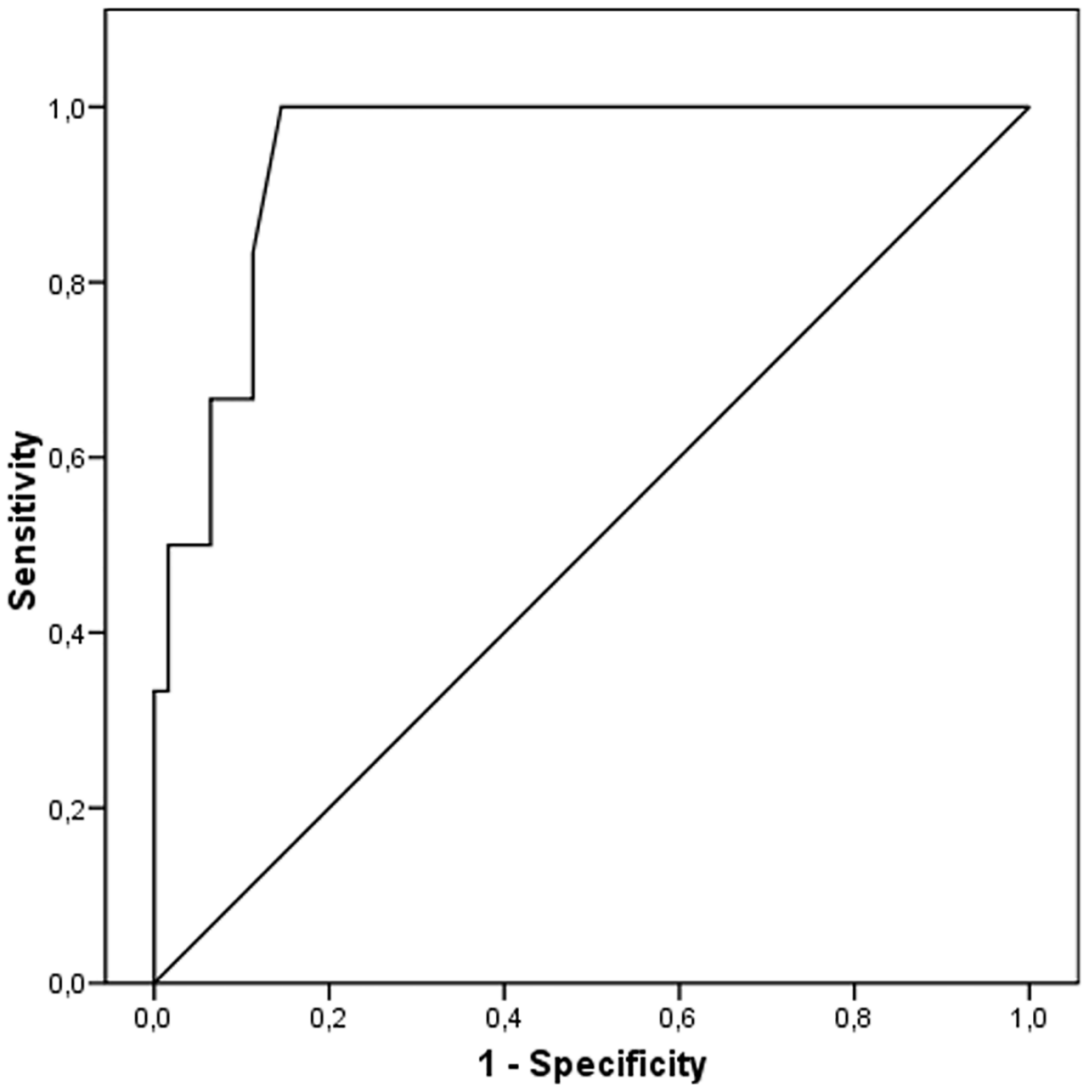
ROC curve on number of VEBs and major cardiac events. ROC curve on number of VEBs and major cardiac events (considered as sudden cardiac death or ICD implantation). AUROC = 0.92, p<0.0001. ROC = receiver operating curve. AUROC = area under ROC.

To study the role of 24h-ECG-Holter in clinical practice, we performed a ROC curve analysis in order to determine the best VEBs threshold capable of identifying patients who died of SCD or in whom an ICD implantation has to be considered. On ROC curve, VEBs >1190/24h showed 100% of sensitivity and 83% of specificity to predict SSc cardiac-related complications (i.e. SCD or ICD-implantation) (AUROC = 0.92,p<0.0001)(Fig 2). Patients with VEBs >1190/24h coincided with those with VEBs >1000/24h. Considering the threshold value of VEBs emerged on ROC curve as prognostic significant, patients with VEBS>1190/24h had higher hs-cTnT levels and a lower LV-EF on echocardiography compared to patients with VEBs <1190/24h; moreover, these patients more frequently presented anti-Scl70 positivity. Eighteen patients (75%) with VEBs>1190/24h presented an increase of hs-cTnT with respect to 43 patients (56.5%) with an increase of hs-cTnT but VEBs<1190/24h, even though this latest difference did not reach the statistical significance (Table 4).

**Table 4:** Clinical characteristics of patients with and without VEBs >1190/24h.

Characteristics	Univariate analysis		
	24 patients with VEBs>1190/24h	76 patients with VEBs <1190/24h	p*
**Age (years), mean±SD**	51.7 ± 16.3	57.5 ± 14.6	0.1
**Disease duration (years), mean±SD**	10.1 ± 9.2	10.3 ± 9.3	0.9
**Anti-Scl70 positivity, n(%)**	15 (62.5)	31 (40.8)	0.05
**Diffuse cutaneous disease, n(%)**	17 (70.8)	38 (50.0)	0.06
**Skin score, mean±SD**	11.9 ± 11.9	8.9 ± 8.4	0.4
**History of digital ulcers, n(%)**	17 (70.8)	42 (55.3)	0.1
**PAH, n(%)**	2 (8.3)	13 (17.1)	0.6
**NT-proBNP values pg/ml, mean±SD**	2804.4 ± 4752.1	719.3 ± 1652.5	0.06
**NT-proBNP >125 pg/ml, n(%)**	16 (66.6)	38 (50.0)	0.1
**Troponin T values ng/ml, mean±SD**	0.09 ± 0.16	0.04 ± 0.07	0.02
**Troponin T >0.014 ng/ml, n(%)**	18 (75.0)	43 (56.5)	0.08
**CK-MB values, ng/ml, mean±SD**	6.5 ± 12.3	3.9 ± 4.8	0.1
LV-EF (%)*, mean±SD	53.1 ± 12.3	62.3 ± 7.4	<0.001
LV-EF <55%*, n(%)	10 (41.7)	9 (11.8)	0.001
**SVEBs, n/24h, n(%)**	1305.6 ± 2205.4	652.3 ± 1702.6	0.006
**SVEBs>1000/24h, n, mean±SD**	9 (37.5)	10 (13.6)	0.003
**Complete RBBB, n(%)**	6 (25.0)	13 (17.1)	0.2
**NSVT, n(%)**	8 (33.3)	3 (3.9)	<0.001
**Palpitations, n(%)**	15 (62.5)	43 (56.5)	0.4
**Dyspnea, n(%)**	14 (58.3)	54 (71.1)	0.5

VEBs = ventricular ectopic beats; SVEBs = supraventricular ectopic beats; n = number; SD = standard deviation; RBBB = right branch bundle block; NSVT = non-sustained ventricular tachycardia; PAH: pulmonary arterial hypertension.

*Comparison between two subgroups; Mann-Whitney non parametric test was used for continuous variables, while Chi-Square test was used for categorical variables.

At univariate analysis, the diffuse cutaneous form, anti-Scl70 positivity, hs-cTnT >0.014 ng/ml, NT-proBNP >125 pg/ml, LV-EF <55% and the presence of RBBB, NSVT or SVBEs >1000/day on 24h-monitoring were associated with VEBs >1190/24h.

At multivariate analysis, the presence of hs-cTnT >0.014 ng/ml (RR:3.1,CI:1.1–9.2,p = 0.04) and of RBBB (RR:5.4, CI:1.3–22.2,p = 0.02) on ECG emerged as independent predictors of VEBs>1190/24h on ECG-Holter.

## Discussion

Our data confirm and support the notion that arrhythmias are common in SSc patients, particularly in those with an increase of hs-cTnT and/or with symptoms suggestive of heart involvement, even if mild and not specific, and represent a risk factor for cardiac complications. Both ventricular and supraventricular arrhythmias, indeed, were present in more than 50% of our SSc patients with presumable heart involvement and the mean number of VEBs was strikingly high; conversely, Holter abnormalities were extremely uncommon in asymptomatic SSc patients without increase of cardiac enzymes. This observation suggests that a careful clinical evaluation associated with a simple and repeatable non-invasive tests determination of hs-cTnT plasma levels should represent the first step in the risk stratification for SCD, considering its ability to detect SSc patients at higher arrhythmic risk and therefore eligible for a comprehensive cardiac evaluations including 24h-ECG Holter. In our cohort of selected SSc patients, the presence of VEBs>1190/24h was found in 24% of patients with suspected cardiac involvement, mainly in those with a hs-cTnT increase and a RBBB on ECG. At multivariate analysis, indeed, the presence of increased hs-cTnT and of RBBB emerged as independent predictors of VEBs>1190/24h on ECG-Holter; this finding leads us to speculate about the prognostic importance of an high-risk association between these warning biomarkers in SSc, taking into account the fact that both hs-cTnT and RBBB are two recently described poor prognostic factors in SSc patients [7,22].

The VEBs cut-off value emerged on ROC curve showed a high sensitivity and specificity in the prediction of our primary end-point: SCD and need for ICD-implantation in SSc-patients without PAH. The presence of VEBs>1190/24h on 24hECG-Holter could be considered a marker of cardiac damage, as revealed by its direct correlation with hs-cTnT levels and inverse correlation with LV-EF on echocardiography, and can help to identify patients with increased risk of major and potentially fatal arrhythmic events and bad cardiac outcome.

### From VEBs to fatal arrhythmias: pathogenetic hypothesis

The transition from VEBs to fatal arrhythmia can be mediated by various triggers, such as an increase of sympathetic tone [23]. This so-called “premature ventricular contractions (PVC)-hypothesis” assumes that VEBs serve as triggers for the initiation of ventricular tachycardia or fibrillation (VT/VF) and that suppression of VEBs protects against SCD by eliminating this triggering function, underlying again the importance of VEBs detection to better risk stratify high risk patients. This concept gained popularity from early observations in coronary care units among patients with acute ischemic events [24,25], and the view of VEBs as primary triggering events for fatal arrhythmias expanded and became ingrained into clinical dogma [23]. An alternative explanation for the observed association between VEBs and the risk for sudden death is reverse causation; that is, presence of frequent VEBs may just be a marker of an underlying severe cardiac disease. Indeed, observational studies have reported that VEBs are associated with poor cardiac outcome in patients with myocarditis [26], and in the general population frequent VEBs are associated with a substantial increase in the risk of sudden and total cardiac death [13].

### Prognosis of SSc patients with heart involvement: 24h ECG-Holter as an additional risk-stratification test

The identification of the importance of frequent VEBs in selected SSc-patients and the finding on ROC curve analysis of a possible VEBs cut-off, in particular, can be a starting point for larger-scale studies to better define cardiac involvement prognostication in scleroderma disease. The early detection of the arrhythmic burden could allow the identification of patients with poor prognosis in order to prevent malignant arrhythmias and sudden cardiac death through prompting therapeutic interventions and a closer follow-up. In this scenario, a number of VEBs>1190/24h appears to have an excellent positive predictive value in the identification of patients at high risk of SCD and therefore eventually candidates for an ICD implantation. In a recent study an ICD was implanted in 10 SSc patients with cardiac involvement and ventricular arrhythmias on 24h ECG-Holter, and analysis of devices after 36 months revealed several episodes of VT in 3 patients (all with a number of VEBs>5000/24h at baseline ECG-Holter), which were promptly reverted by electrical shock delivery, underlying again the prognostic importance of Holter abnormalities [27] and the clinical significance of primary prevention of SCD in selected SSc populations. Currently, guidelines for the management of cardiac arrhythmias and for the use of ICD to prevent SCD mainly target patients who have already survived a SCD or a life-threatening arrhythmic event, or those with a severe underlying heart disease; thus, it’s primarily a secondary prevention. Conversely, in certain populations the clinical-benefit and the cost-effectiveness of ICD therapy to prevent SCD appear to be marginal and ejection fraction is the primary factor used to select patients for ICD therapy [13,20] and for primary prevention. In patients who fall into the so-called “borderline” areas, such as those with ejection fractions between 30% to 40% or those with class I heart failure, additional risk-stratification tests may be useful when applied on an individual basis. Nowadays, there are no adequate available data to routinely recommend additional risk stratification techniques. In that view, other diagnostic tests examining both fixed and transient factors that may predispose to sudden death, and among these 24h-ECG-Holter, could potentially ameliorate patient selection for ICD-based approach and thus improve clinical benefit and patient outcome, especially in specific diseases at high risk for arrhythmic cardiac mortality. The observation that among the 7 patients who met the primary end-point in our cohort one of them had a preserved LV-EF on echocardiography and another one fell in the borderline area, but all of them presented at least 1190 VEBs per day, underlines the aforementioned unmet need and strengthens once again the prognostic role of VEBs and the clinical usefulness of 24h ECG-Holter analysis.

Furthermore, even considering other previously described prognostic factors and already identified arrhythmic abnormalities on ECG with prognostic significance in SSc patients [3,7], we should note that in our cohort of selected SSc patients, frequent VEBs were found in all patients with bad outcome while none of the patients without frequent VEBs died or developed major arrhythmic complications. Conversely, none of demographic or disease-related characteristics and previously reported ECG abnormalities emerged as so peculiar in predicting poor outcome.

### Arrhythmias in SSc: a smouldering fire

The knowledge that arrhythmias are a frequent event and represent a major cause of death in SSc dates back more than thirty years ago, but nowadays it seems to be underestimated by clinicians. Post-mortem analysis by Follansbee revealed that 5% of death were due to SCD, that was associated with the presence of skeletal myositis and ventricular arrhythmias, both frequent conditions during the disease, even among asymptomatic patients [4,5]. Ventricular ectopy, in particular, occurred overall in 67% of cases in a previous prospective multicenter study of 183 SSc patients (maily females with a diffuse disease and asymptomatic for palpitations) studied with 24-hour ambulatory ECG and followed for 33 months, and was strongly correlated with total mortality and sudden death; the presence of ventricular tachycardia and of VEBs>1000/24h, in particular, was associated with a relevant increase of mortality [6]. In our study the prognostic role of frequent VEBs emerged in patients without PAH, while previously the role of VEBs and SCD were reported in patients with evidence of severe pulmonary involvement and PAH [6]. PAH, in particular, is a condition in which cardiac arrhythmias are important contributors to morbidity and mortality and the SCD accounts alone for 28% deaths [9]. In the study by Kostis and co-workers, indeed, 8 of the 12 patients died for SCD had clinical severe pulmonary involvement, including four with PAH on RHC [6]. Whilst recognizing the importance of this study, the ventricular arrhythmias were not correlated with other markers of cardiac damage as hs-cTnT or echocardiographic parameters, among all LV-EF. To our knowledge this is the first study in which ventricular arrhythmias are studied along with other cardiac parameters to identify patients at risk of a bad outcome.

Importantly, the prevalence and the prognostic significance of ventricular arrhythmias in SSc patients with direct cardiac involvement, i.e. unrelated to pulmonary disease, is not so well characterized, despite a growing interest in this topic in the recent years. The notion that arrhythmias are associated with a dismal prognosis, in fact, was emphasized by a recent report from GENISOS cohort [3]. However, despite its remarkable epidemiological impact and the reported prognostic importance of cardiac arrhythmias on standard ECG, in this multicenter study the single prognostic significance of different abnormalities on baseline ECG (i.e. VEBs, SVBEs, conduction delays, rhythm alteration) is lacking. To date, few previous studies focused on prognostic significance of standard ECG or ECG-Holter abnormalities in scleroderma patients and no clear predictors of cardiac involvement and outcome emerged. According to our results, RBBB is a recently recognized independent predictor of mortality and should be considered a marker of disease severity in SSc. Indeed, the study by Draeger on 256 patients showed that a complete RBBB on ECG, present in 7 patients (2.6%), predicted a higher risk of mortality; the authors confirmed, moreover, the high prevalence of rhythm and conduction abnormalities in the course of Scleroderma [7].

In our monocentric study, we focused our attention on 24h-ECG Holter monitoring and on VEBs, to study a specific risk factor for cardiac death in selected SSc patients; even if preliminary and limited to a small sample size, our results could shed a new light into the management of SSc patients with presumable cardiac involvement and possibly help to define a risk-stratification algorithm that allows a prompt therapeutic and life-saving intervention in high risk patients.

### The arrhythmic burden in SSc: pathogenetic mechanisms and role of myocardial inflammation

The high frequency of arrhythmias is historically related to patchy myocardial fibrosis [28], that provides an ideal substrate for tachyarrhythmias dependent on re-entrant circuits by disrupting the normal electrical connectivity of cardiac tissue [29]. However, an important role in arrhythmogenesis could also be played by the inflammatory burden (in particular in the presence of myocarditis) [30], as well as by the production of anti-β-adrenoceptors antibodies, which have been found in patients with dilated cardiomyopathy and with "idiopathic arrhythmias” [31], and by the presence of autonomic dysfunction, which is extremely common in SSc, starting early in the disease process and possibly even preceding the development of fibrosis [32]. There are, indeed, clinical and experimental evidence of a link between the propensity for life threatening arrhythmias and both myocarditis [23,26,30] and sympatho-vagal imbalance [33]. This latter association is based on the notion that increased sympathetic activity appears to be pro-arrhythmic, whereas β-blocker therapy and enhanced parasympathetic tone counteract this arrhythmogenic effect. Abnormalities in the baseline parasympathetic tone, as represented by reduced heart rate variability (HRV), identify patients at a high risk for developing ventricular tachycardia and SCD [33,34].

The pathogenesis of SSc-related heart disease and arrhythmic burden is still controversial and poorly understood. The vascular mechanism hypothesis is traditionally the most credited, dating back to necropsy studies and suggesting that myocardial fibrosis might be caused by ischemic necrosis and reperfusion damage following intermittent vascular spasms [28]. In our study, all seven patients who met the primary end-point had a history of digital ulcers, and this finding could indirectly strengthens the vascular hypothesis. Furthermore, in a recent study on an Italian cohort of 20 SSc patients without cardiac involvement, QTc interval prolongation showed a linear correlation with clinical variables secondary to vascular complications of the disease, indirectly suggesting that SSc patients with specific features as late capillaroscopic pattern and presence of digitals ulcers may have a particularly high risk of developing life-threatening arrhythmias [35]. However, in our larger cohort of selected SSc patients with presumable cardiac involvement, QTc was found to be prolonged in 11 of them and was correlated neither with major arrhythmic complications nor with a higher frequency of digital ulcers.

Recent studies using delayed-enhancement (DE) cardiac magnetic resonance (CMR), nevertheless, seem to discount the vascular mechanism hypothesis, since fibrosis was found to have non-coronary distribution and to be midwall with predominantly linear pattern [36,37]. The same pattern follows myocardial lymphocyte infiltration in idiopathic dilated cardiomyopathy and inflammatory cardiomyopathies [38,39]. Inflammatory heart involvement has been occasionally reported in SSc patients [40,41]; in the present study eight patients, five of whom with DE on CMR, underwent endomyocardial biopsy (data not shown), and in all, a histologically-proven myocarditis with endothelial up-regulation of adhesion molecules and intense tissue infiltration by activated/memory T-helper lymphocytes was described [30]. Consistent with our previous data, Mueller and coauthors recently described the clinical characteristics, histopathological findings and outcome of 26 SSc patients with clinical phenotype suggestive of cardiac involvement and they associated the degree of cardiac inflammation on endomyocardial biopsy, detected in 96.2% of cases, with patient outcome [42]. Despite such increasing interest, however, the occurrence of myocarditis in SSc heart disease is still likely mis-diagnosed and underestimated.

These data and the aforementioned findings by DE-CMR, as well as the correlation described in the present study between VEBs and hs-cTnT levels, suggest that myocarditis could have an important role in the pathogenesis of SSc-related heart disease and of its arrhythmic complications.

### Study limitations

Our monocentric study has some limitations; the major one is the number of patients enrolled and the low percentage of major arrhythmic events in our cohort, likely explaining the failure of any variable to emerge as an independent predictor of SCD on multivariate analysis. Our patients, however, underwent a 24h-ECG Holter monitoring at baseline; given the lack of repeated ECG Holter monitoring or implantable event recorders, some arrhythmic events might therefore have been missed.

On the other hand we selected a homogeneous cohort of SSc-patients with suspected cardiac involvement, i.e. with new onset of symptoms suggestive of cardiac involvement (such as dyspnea, palpitations, chest pain, and/or signs of heart failure) and/or with an increase of cardiac enzymes.

The number of patients who underwent a complete invasive assessment consisting of coronary angiography and endomyocardial biopsy in our cohort and the lack of autopsy data on the 5 sudden death cases restrict the opportunity to clarify the pathogenetic mechanisms underlying the SSc arrhythmic burden in all cases, as well as the opportunity to certainly rule out the presence of coronary artery disease in all patients. The contribution of autonomic dysfunction cannot be addressed because HRV was not assessed in all patients. Certainly, noninvasive HRV evaluation might be beneficial when added to the clinical and laboratory assessments in detecting high-risk patients, and may allow for implementation of preventive measures and initiation of appropriate therapy early in the course of the disease.

Based on these limitations, our data confirm, in patients without pulmonary hypertension, the data of the previous old prospective study and, most importantly, suggest that the cut-off value of VEBs that emerged on ROC curve, could be the “warning biomarker “of major cardiac risk in SSc patients populations.

## Conclusions

Arrhythmias are frequent in SSc patients with signs and symptoms suggestive of heart involvement, particularly in those with increase of hs-cTnT and RBBB on ECG; VEBs, in particular, correlate with cardiac damage and identify patients at high risk of major and life-threatening arrhythmic complications. The high prevalence and the prognostic value of VEBs on 24h-ECG-Holter, rarely recorded on standard ECG, highlight the need for a complete assessment in selected SSc-patients, also at the early stages of the disease. The early detection of the arrhythmic burden could allow the identification of patients with poor prognosis in order to prevent malignant arrhythmias through prompting therapeutic interventions and a closer follow-up. In this view, our data suggest 24h-ECG Holter as an additional risk-stratification technique for selection of SSc patients at high-risk of SCD, in whom an ICD-implantation for primary prevention could represent a potential life-saving intervention. Our preliminary data can be a starting point for larger-scale studies with a longer follow-up, to better define cardiac involvement prognostication in scleroderma disease.

## Supporting Information

S1 Appendix Further information on “patients and methods” paragraph. (DOC)

S1 Table ECG-Holter findings in our cohort of 100 selected SSc patients with presumable heart involvement and in 15 asymptomatic SSc patients. Pts: patients; n: number; SD: standard deviation. HR = heart rate; bpm = beats per minute; *SVEBs: number of supraventricular ectopic beats during 24h evalutation; ^†^VEBs: number of ventricular ectopic beats during 24h evaluation; ^§^patients with at least 100 VEBs or SVEBs during 24h; **this percentage was calculated on patients with VEBs.
(DOCX)
