# Supplementary material for: Prognostic Role of Ventricular Ectopic Beats in Systemic Sclerosis: A Prospective Cohort Study Shows ECG Indexes Predicting the Worse Outcome
Source: PLoS One. 2016 Apr 21;11(4):e0153012. doi: 10.1371/journal.pone.0153012 (PMC4839708; doi:10.1371/journal.pone.0153012)
Supplement: S1 Appendix — (DOC) [file pone.0153012.s001.doc]

**S1 Appendix: supplemental file**

A comprehensive assessment of disease characteristics and organ involvement was performed and disease severity index was calculated according to Medsger scale in all patients [1,2]. Data on cardiovascular risk factors such as arterial hypertension or diabetes mellitus, smoking history, body mass index (BMI) and serum levels of total cholesterol, high-density-lipoprotein (HDL), fasting glucose and triglycerides were available for all patient cohort.

ANA(antinuclear antibodies) were determined by indirect immunofluorescence using Hep2-cells as substrates and autoantibody specificities were assessed by ELISA [3].

Cardiac enzymes [cardiac troponin-T(cTnT) and CK-MB], total creatine-phosphokinase (CPK) and NT-proBNP values were obtained in all patients within two weeks of ECG-Holter monitoring; cTnT was measured using the quantitative electrochemiluminescence immunoassay (Roche Diagnostic, Mannheim, Germany). The concentration of NT-proBNP was measured by sandwich immunoassay on an Elecsys-2010 instrument (Roche Diagnostic, Basel, Switzerland).

Pulmonary Function Tests (PFTs) were performed in all patients to define forced vital capacity (FVC) and carbon monoxide diffuse capacity (DLCO), as described elsewhere [4,5]. FVC<80% of predicted values with normal forced expiratory volume in one second(FEV1)/FVC ratio defined a restrictive lung disease [4-6]. Lack of lung progression was defined as the absence of either new relative reduction more than 10% in FVC and/or more than 15% in the DLCO or further extension of ground glass opacities and/or honeycombing pattern at lung high-resolution computed tomography [7,8].

The presence of coronary artery disease was ruled-out by medical history and clinical data, by findings on 12-leads ECG and 2D-echocardiographic Doppler examination and, in 23 patients, by coronary angiography. Given the invasive nature of this latest exam, it was not routinely performed in all patients but based on clinical cardiologist judgement. When clinically appropriate, an exercise myocardial perfusion scintigraphy was also performed.

None of the patients had hyperthyroidism or chronic obstructive pulmonary disease, both conditions possibly associated with the presence of arrhythmias.

BMI was categorized into 4 classes, i.e <18 kg/m^2^(underweight), 18-25 kg/m^2^(normal weight), 25-30 kg/m^2^(overweight), and >30 kg/m^2^(obese). Hypertriglyceridemia was defined as the presence of triglycerides above 150mg/dl, hypercholesterolemia as total cholesterol above 200mg/dl, low-HLD when below 40mg/dl in men and below 50mg/dl in women.

The analysis of individual cardiovascular risk factors highlighted that 46 patients (46%) presented lipid abnormalities, 19 (19%) had a diagnosis of arterial hypertension, adequately controlled at study entry, and 7 (7%) diabetes mellitus. Ten patients (10%) were current smokers and 12 (12%) were ex-smokers for more than 10 years. The mean BMI was 23.7±4.5 kg/m^2^ and the prevalence of obesity was 8%. According to BMI, 66 patients (66%) were normal-weight, 24 (24%) were overweight, 8 (8%) were obese, while only 2 patients were underweight.

**References**

1. G Valentini, W Bencivelli, S Bombardieri, S D’Angelo, A Della Rossa, A J Silman, C M Black, L Czirjak, H Nielsen, P G Vlachoyiannopoulos. European Scleroderma Study Group to define disease activity criteria for systemic sclerosis. III. Assessment of the construct validity of the preliminary activity criteria. Ann Rheum Dis 2003;62:901–903.
2. Medsger TA , Silman AJ, Steen VD, Black CM, Akesson A, Bacon PA, et al.A disease severity scale for systemic sclerosis. Development and testing. J Rheumatol 1999;26:2159–67.
3. De Santis M, Bosello S, La Torre G, Capuano A, Tolusso B, Pagliari G, Pistelli R, Danza FM, Zoli A, Ferraccioli G. Functional, radiological and biological markers of alveolitis and infections of the lower respiratory tract in patients with systemic sclerosis. Respir Res 2005;17:6,96.
4. American Thoracic Society. Standardization of Spirometry-1994 Update. Am J Respir Crit Care Med 1995;152:1107-1136.
5. American Thoracic Society. Single breath Carbon Monoxide Diffusing Capacity (Transfer Factor). Recommendation for a standard technique-1995 Update. Am J Respir Crit Care Med 1995;152:2185-2198.
6. Egan JJ, Martinez FJ, Wells AU, Williams T**.** Lung function estimates in idiopathic pulmonary fibrosis: the potential for a simple classification. Thorax 2005;60:270-3.
7. Behr J, Furst DE. Pulmonary function tests. Rheumatol 2008;47:65-7.
8. Bosello S, De Luca G, Rucco M, Berardi G, Falcione M, Danza FM, Pirronti T, Ferraccioli G. Long-term efficacy of B cell depletion therapy on lung and skin involvement in diffuse systemic sclerosis. Semin Arthritis Rheum 2015;44(4):428-36.
