# Supplementary material for: Prognostic Role of Ventricular Ectopic Beats in Systemic Sclerosis: A Prospective Cohort Study Shows ECG Indexes Predicting the Worse Outcome
Source: PLoS One. 2016 Apr 21;11(4):e0153012. doi: 10.1371/journal.pone.0153012 (PMC4839708; doi:10.1371/journal.pone.0153012)
Supplement: S1 Table — Pts: patients; n: number; SD: standard deviation. HR = heart rate; bpm = beats per minute; *SVEBs: number of supraventricular ectopic beats during 24h evalutation; †VEBs: number of ventricular ectopic beats during 24h evaluation; §patients with at least 100 VEBs or SVEBs during 24h; **this percentage was calculated on patients with VEBs. (DOCX) [file pone.0153012.s002.docx]

| **24h ECG-Holter abnormalitis** | **100 SSc patients with heart involvement** | **15 asymptomatic SSc patients** | **p** |
| --- | --- | --- | --- |
| Pts with any alteration on 24h ECG-Holter, n (%) | 56 (56) | 4 (26.7) | 0.032 |
| Sinus rhythm, n (%) | 96 (96) | 15 (100) | 0.6 |
| Phases of idioventricular rhythm, n (%) | 2 (2) | 0 (0) | 0.7 |
| Phases of bigeminal rhythm, n (%) | 9 (9) | 0 (0) | 0.3 |
| Atrial fibrillation, n (%) | 4 (4) | 0 (0) | 0.6 |
| I° degree atrio-ventricular block, n (%) | 4 (4) | 0 (0) | 0.6 |
| Right bundle branch block, n (%) | 19 (19) | 0 (0) | 0.06 |
| Left bundle branch block, n (%) | 4 (4) | 0 (0) | 0.6 |
| Mean HR, bpm (mean ± SD) | 79.4 ± 10.2 | 76.8 ± 8.8 | 0.5 |
| Maximum HR, bpm (mean ± SD) | 131.7 ± 23.3 | 140.6 ± 46.1 | 0.9 |
| Minimum HR, bpm (mean ± SD) | 54.8 ± 11.2 | 52.1 ± 6.3 | 0.4 |
| SVEBs^*^, n/24h (mean ± SD) | 798.9 ± 1835.6 | 47.5 ± 87.6 | 0.004 |
| VEBs^†^, n/24h (mean ± SD) | 2046.1 ± 6027.8 | 63.9 ± 136.6 | 0.028 |
| Pts with VEB>1000/24h, n (%) | 24 (24) | 0 (0) | 0.023 |
| Pts with SVEB>1000/24h, n (%) | 19 (19) | 0 (0) | 0.05 |
| Pts with polymorphic VEB, n (%) | 11 (26.2)^**^ | 0 (0) | 0.06 |
| Supraventricular tachycardia, n (%) | 14 (14) | 4 (26.7) | 0.2 |
| Non sustained ventricular tachycardia, n (%) | 11 (11) | 0 (0) | 0.18 |

**S1 Table.** ECG-Holter findings in our cohort of 100 selected SSc patients with presumable heart involvement and in 15 asymptomatic SSc patients.

Pts: patients; n: number; SD: standard deviation. HR= heart rate; bpm= beats per minute; *SVEBs: number of supraventricular ectopic beats during 24h evalutation; ^†^VEBs: number of ventricular ectopic beats during 24h evaluation; ^§^patients with at least 100 VEBs or SVEBs during 24h; ^**^this percentage was calculated on patients with VEBs.
